# Diagnostic Approaches and Established Treatments for Adult T Cell Leukemia Lymphoma

**DOI:** 10.3389/fmicb.2020.01207

**Published:** 2020-06-19

**Authors:** Kunihiro Tsukasaki, Ambroise Marçais, Rihab Nasr, Koji Kato, Takahiro Fukuda, Olivier Hermine, Ali Bazarbachi

**Affiliations:** ^1^Department of Hematology, International Medical Center, Saitama Medical University, Saitama, Japan; ^2^Institut Imagine - INSERM U1163, Necker Hospital, University of Paris, Paris, France; ^3^Department of Hematology, Necker Hospital, University of Paris, Assistance Publique Hôpitaux de Paris, Paris, France; ^4^Department of Anatomy, Cell Biology and Physiological Sciences, Faculty of Medicine, American University of Beirut, Beirut, Lebanon; ^5^Department of Hematology, Oncology and Cardiovascular Medicine, Kyushu University Hospital, Fukuoka, Japan; ^6^Department of Hematopoietic Stem Cell Transplantation, National Cancer Center Hospital, Tokyo, Japan; ^7^Department of Internal Medicine, Faculty of Medicine, American University of Beirut, Beirut, Lebanon

**Keywords:** adult T-cell leukemia-lymphoma (ATL), treatment, zidovudine (AZT), allogeneic hematopoietic stem cell transplantation (allo-HSCT), chemotherapy

## Abstract

Adult T-cell leukemia-lymphoma (ATL) is a distinct mature T-cell malignancy caused by human T-cell leukemia/lymphotropic virus type I (HTLV-1) endemic in some areas in the world. HTLV-1 transmits through mother-to-child infection via breastfeeding, sexual intercourses, and blood transfusions. Early HTLV-1 infection, presumably through mother’s milk, is crucial in developing ATL. The estimated cumulative risk of the development of ATL in HTLV-1 carriers is a few percent after transmission from their mothers. The median age of ATL onset is about 70 in Japan and is now rising, whereas an overall mean age in the mid-forties is reported in other parts of the world. ATL is classified into four clinical subtypes (acute, lymphoma, chronic, and smoldering) defined by organ lesions and LDH/calcium values. In aggressive ATL (acute, lymphoma or unfavorable chronic types) and indolent ATL (favorable chronic or smoldering types), intensive chemotherapy followed by allogeneic hematopoietic stem cell transplantation and watchful waiting until disease progression has been recommended, respectively, in Japan. Based on a worldwide meta-analysis and multiple other retrospective studies, the antiviral combination of interferon alpha (IFN) and zidovudine (AZT) is recommended in many parts of the world in acute, chronic, and smoldering ATL whereas patients with the lymphoma subtype are treated with chemotherapy, either alone or combined with AZT/IFN. Several new agents have been approved for ATL by the Pharmaceutical and Medical Devices Agency (PMDA) after clinical trials, including an anti-CC chemokine receptor 4 monoclonal antibody, mogamulizumab; an immunomodulatory agent, lenalidomide; and an anti-CD30 antibody/drug conjugate, brentuximab vedotin.

## Introduction

Adult T-cell leukemia–lymphoma (ATL) is a mature, peripheral T-cell malignancy of Treg/Th2 phenotype associated with human T-cell leukemia/lymphotropic virus type I (HTLV-1) infection with a high frequency of expression of CD3/CD4/CD25/CCR4 and FoxP3 in about half of the cells (Takatsuki, 1994; Tsukasaki et al., 2009; Ohshima et al., 2017; Cook Lucy et al., 2019). In fresh ATL cells, although expression of the virus, including Tax, comes just after an *in vitro* culture, the sites of proviral integration into the host genome are random, and abnormalities in the chromosome/genome are complex (Takatsuki, 1994; Tsukasaki et al., 2009; Kataoka et al., 2015; Ohshima et al., 2017; Cook Lucy et al., 2019). Thus, ATL is a unitary disease entity that is associated with HTLV-1 with diverse molecular and clinical features. Worldwide, at least 5–10 million individuals are infected with HTLV-1; however, the highly endemic regions of HTLV-1 are limited to Japan, sub-Saharan Africa, South America, the Caribbean, central Australia, the Middle East, and Romania (Takatsuki, 1994; Cook Lucy et al., 2019). In Japan by estimation, there are one million HTLV-1 carriers, 4,000 new HTLV-1 infection annually mainly through sexual transmission, and 1,000 deaths from ATL annually (Iwanaga et al., 2012; Satake et al., 2012; Satake et al., 2016). Recently, both HTLV-1 carriers and ATL patients are aging and spreading outside or spreading beyond the endemic southwestern region of Japan, throughout the country, particularly in the greater Tokyo metropolitan area (Satake et al., 2012; Nosaka et al., 2017). It has been reported that the age of patients with ATL in areas outside Japan is younger, with an overall mean age in the mid-forties (Yamaguchi and Takatsuki, 1993). Only a few percent of HTLV-1 carriers, who were transmitted through breastfeeding, develop ATL indicating multistep leukemogenesis (Okamoto et al., 1989; Tajima, 1990). The diversity in clinical features, prognosis, and natural history of patients with this disease resulted in its subtype classification into four categories, acute, lymphoma, chronic, and smoldering types defined by organ involvement, LDH, and calcium values (Shimoyama, 1991). ATL is refractory to chemotherapy, but sensitive to allogeneic hematopoietic stem cell transplantation (allo-HSCT) and to interferon (IFN)/zidovudine (AZT) therapy (Tsukasaki et al., 2009). Recently, several new agents have been approved by PMDA (Cook Lucy et al., 2019). This review will focus on molecular and clinical pathophysiology and established treatments of ATL.

## Diagnosis, Subtype Classification, Prognostic Factors, and Prognostic Index of ATL

After HTLV-1 was revealed to be associated with ATL, it was found that ATL shows a marked diversity in its clinical manifestations (Shimoyama, 1991; Takatsuki, 1994; Ohshima et al., 2017). HTLV-1 provirus is monoclonally integrated in ATL cells (Ohshima et al., 2017). Molecular analysis of the proviral integration is recommended when possible (Tsukasaki et al., 2009). Either Southern blotting or PCR for the virus can be used to detect the presence of HTLV-1 integration, whereas the latter is better for quantification. However, the monoclonal integration is also shown in some HAM/TSP patients and HTLV-1 carriers with high proviral load in peripheral blood mononuclear cells (PBMCs) (Tsukasaki et al., 2009). Clinically, the diagnosis of ATL is made based on seropositivity for HTLV-1 and histologically and/or cytologically proven peripheral T cell lymphoma (PTCL), although rare cases of other PTCL developing in HTLV-1 carriers have been reported (Tsukasaki et al., 2009).

ATL cases have been subdivided into four distinct clinicopathologic entities: acute, lymphoma, chronic, and smoldering types (Shimoyama, 1991). The recognition of the four clinical subtypes is important in understanding the natural history, clinical features, treatment strategy, and leukemogenesis of ATL. On the basis of a nationwide survey of 854 patients with ATL who were diagnosed between 1983 and 1987 in Japan, the Lymphoma Study Group proposed diagnostic criteria for the four clinical subtypes based on the sites of organ infiltration, presence, absence, and degree of leukemic manifestation, and high LDH/calcium levels (Table 1) (Shimoyama, 1991).

**TABLE 1:** Diagnostic criteria for clinical subtypes of adult T-cell leukemia–lymphoma.

	Smoldering	Chronic	Lymphoma	Acute
Anti-HTLV-1 antibody	+	+	+	+
Lymphocyte (×10^3^/μUL)	< 4	≥ 4^‡^	< 4	*
Abnormal T lymphocytes	≥ 5%^¶^	+^§^	≤ 1%	+^§^
Flower cells with T-cell marker	^†^	^†^	No	+
LDH	≤ 1.5 N	≤2N	*	*
Corrected Ca^2+^ (mEq/L)	< 5.5	<5.5	*	*
Histology-proven lymphadenopathy	No	*	+	*
Tumor lesion				
Skin and/or lung	*	*	*	*
Lymph node	No	*	Yes	*
Liver	No	*	*	*
Spleen	No	*	*	*
Central nervous system	No	*	*	*
Bone	No	No	*	*
Ascites	No	No	*	*
Pleural effusion	No	No	*	*
Gastrointestinal tract	No	No	*	*

*HTLV-1, human T-lymphotropic virus type I; LDH, lactate dehydrogenase; N normal upper limit. ^∗^No essential qualification except terms required for other subtype(s). ^†^Typical “flower cells” may be seen occasionally. ^§^ If the proportion of abnormal T lymphocytes is less than 5% in peripheral blood, a histologically proven tumor lesion is required. From Shimoyama M, Members of the Lymphoma Study Group (1984–1987): Diagnostic criteria and classification of clinical subtypes of adult T-cell leukemia-lymphoma. Br J Haematol 1991;79:428.*

The acute type shows a rapidly progressive clinical course and major clinical features of ATL: leukemic manifestation, systemic lymphadenopathy, hepatosplenomegaly, skin lesion, hypercalcemia, and the infiltration of other organs (central nervous system, gastrointestinal tract, etc.). In addition, the diagnosis of the acute ATL is based on the exclusion of the other three subtypes. The symptoms and signs include abdominal pain, diarrhea, ascites, jaundice, pleural effusion, productive cough, fever, and unconsciousness because of organ involvement, hypercalcemia, and/or opportunistic infections. The smoldering type shows an indolent clinical course and only a small percentage of leukemic cells, but it also can include skin and lung involvement without hypercalcemia and high LDH value (≤× 1.5 upper normal limit). The chronic type, lymphocytosis with a high percentage of leukemic cells, is occasionally associated with skin and lung involvement, lymphadenopathy, and hepatosplenomegaly without hypercalcemia and high LDH value (≤× 2 upper normal limit) and undergoes an indolent or progressive clinical course. The lymphoma type includes patients who present with the manifestations of aggressive nodal non-Hodgkin lymphoma (NHL) without circulating malignant cells in the peripheral blood and shows a rapidly progressive clinical course.

Because leukemic manifestation that means stage IV is frequent in both indolent (chronic/smoldering) and aggressive (acute) ATL, the clinical subtype is more prognostic than the Ann Arbor stage in ATL (Shimoyama, 1991). Laboratory findings also depend on the clinical subtype of ATL. Leukocytosis is found among patients with the chronic subtype and a portion of those with the acute and smoldering subtypes at presentation, exhibiting characteristic abnormal lymphoid cells with markedly lobulated, highly indented, or lobulated nuclei with condensed chromatin, small or absent nucleoli, and an agranular cytoplasm, termed flower cells. Most patients with the acute or lymphoma subtype of ATL have elevated serum LDH levels. Hypercalcemia with/without bone lesion is also frequent in acute or lymphoma subtypes. By definition, chronic and smoldering subtypes are without hypercalcemia and high LDH value (≤× 2 and × 1.5 upper normal limit, respectively). The serum level of soluble interleukin-2 receptor (sIL-2R), as well as LDH, is suggested to be a useful marker for the differential diagnosis of ATL, for evaluating the clinical aggressiveness of the disease and for monitoring the response to treatment (Kamihira et al., 1994).

The swollen lymph nodes and extranodal lesions in patients with ATL show diffuse NHL of various histologic subtypes, including pleomorphic, large cell, mixed cell, or medium-sized cell types. Histological differential diagnosis of ATL according to the WHO classification includes PTCL not otherwise specified (NOS), anaplastic large cell lymphoma (ALCL), angioimmunoblastic T-cell lymphoma (AITL), mycosis fungoides (MF)/Sezary syndrome (SS), and Hodgkin lymphoma (Ohshima et al., 2017). ATL cutaneous lesions have varied presentations and may be similar to those of MF. Although some lesions have an aggressive course, the majority of these lesions are characterized by an indolent course. Accordingly, and especially where HTLV-1 is endemic, HTLV-1 serology and proviral analysis should be performed to differentiate ATL from cutaneous T cell lymphoma (CTCL) including MF and PTCL (Ohshima et al., 2017; Cook Lucy et al., 2019). Lately, an extranodal primary cutaneous variant was proposed in ATL lymphoma subtype. This new variant is characterized by an aggressive course with primary cutaneous lesions. Macroscopic findings are mostly nodulotumoral. Pathology shows pleomorphic, medium, or large-size cells presenting with noticeable perivascular infiltration and scarce epidermotropism indicative of high-grade T-cell lymphoma diagnosis (Tsukasaki et al., 2014; Cook Lucy et al., 2019).

## Therapeutic Strategies of ATL Based on Its Clinical Subtypes

### Natural Course

ATL most often pursues the prototypic acute course; however, approximately one-fourth of patients show a more indolent course (chronic and smoldering types), with the disease limited predominantly to the peripheral blood and/or skin (rarely in the lung). These patients might experience multiple infections but can remain free of disease progression for many years (Shimoyama, 1991). These indolent diseases frequently progress to devastating acute- or lymphoma-type ATL, an event that is sometimes called the crisis. Some studies have reported that various kinds of infectious episodes might predispose individuals to the transformation from an indolent to an aggressive disease course (Tsukasaki et al., 2009; Cook Lucy et al., 2019). Other studies revealed that the presence of genetic alterations such as tumor suppressor abnormalities and aneuploidy in leukemic cells in chronic and smoldering types was associated with a poor prognosis (Tsukasaki et al., 2009). Recently, sIL-2R levels were identified as an independent prognostic factor for chronic/smoldering-type ATL. The utility of sIL-2R would require further validation in prospective studies (Katsuya et al., 2017).

### Watchful Waiting

Most patients with ATL are not curable with current treatment modalities, even at the early stage of the disease. In addition, no treatment has been shown to prevent progression to a more aggressive disease. Therefore, in Japan, patients with favorable chronic- or smoldering-type ATL have been watched carefully without chemotherapy, excluding topical therapy for cutaneous lesions, for signs of progression to unfavorable chronic, acute, or lymphoma-type ATL (Tsukasaki et al., 2009). However, the long-term follow-up of such patients revealed that the prognosis was worse than expected, i.e., as compared to that of patients in non-advanced chronic lymphocytic leukemia with the mean survival time (MST) of 5.3 years without plateau in the overall survival (OS) curve (Takasaki et al., 2010).

### IFN/AZT

The efficacy of the combination of zidovudine (AZT) and interferon-alpha (IFN) in ATL was initially reported in 2 phase II studies (Gill et al., 1995; Hermine et al., 1995; Bazarbachi and Hermine, 1996). High response rates were described, mostly in previously untreated newly diagnosed acute ATL, although the response duration was relatively short (Tobinai et al., 1995). The significant efficacy of this AZT/IFN combination was confirmed in France on 19 newly diagnosed ATL patients and in the UK on 15 ATL patients (Matutes et al., 2001; Hermine et al., 2002).

The results of a worldwide meta-analysis on ATL survival between 1995 and 2008 (Bazarbachi et al., 2010) confirmed the efficacy of this antiviral combination, which became a standard of care in many parts of the world. This study included 254 ATL patients (116 acute, 18 chronic, 11 smoldering, and 100 lymphoma) and compared first-line antiviral treatment (AZT/IFN) given alone, to chemotherapy alone or followed by antiviral therapy. First-line antiviral therapy significantly improved 5-year OS to 46% as compared to 20% and 12%, respectively, for patients who received first-line chemotherapy alone or followed by antiviral therapy. In subgroup analysis, survival benefit from first-line antiviral therapy alone was restricted to leukemic subtypes of ATL (smoldering, chronic, and acute) but not ATL lymphoma. In the indolent forms of ATL (chronic and smoldering subtypes), an impressive 5-year survival of 100% was described in patients treated with antiviral therapy. In acute ATL, AZT/IFN given alone significantly improved 5-year OS from 10% to 28% with an outstanding 5-year survival of 82% for acute ATL patients who achieved complete remission with antiviral therapy. Unfortunately, in the ATL lymphoma subtype, results were dismal with first-line AZT/IFN and no patient survived 5 years. Importantly, a multivariate analysis confirmed the survival benefit of ATL patients treated with first-line AZT/IFN (HR 0.47; 95% CI 0.27–0.83; *p* = 0.021).

Another retrospective study from the UK confirmed the efficacy of AZT and IFN when combined with chemotherapy. This study included 73 patients with aggressive ATL subtypes (44 lymphoma and 29 acute) (Hodson et al., 2011). A response rate of 81% was achieved in patients who received AZT/IFN combined with chemotherapy as compared to 49% for patients who received chemotherapy alone. This resulted in a doubled median progression-free survival (PFS) (8 versus 4 months). Similarly, AZT/IFN combined with chemotherapy significantly improved median OS in acute ATL (*p* = 0.008) and in ATL lymphoma (*p* = 0.001). Finally, multivariate analysis revealed that exposure of patients with aggressive ATL to AZT/IFN at any time significantly improved OS (hazard ratio 0.23; 95% CI, 0.09 to 0.6; textitp = 0.002).

Recently, a US retrospective study (Malpica et al., 2018) reported 195 patients with ATL (96 lymphoma, 80 acute, 7 unfavorable chronic, 5 favorable chronic, 3 smoldering, and 4 unclassified) diagnosed between 1987 and 2016. Median OS was not reached for unfavorable chronic ATL, 72 months for favorable chronic/smoldering ATL, 10.2 months for patients with ATL lymphoma, and 4.1 months for acute. 4-year OS was 83%, 60%, 4%, and 10%, respectively. Patients treated with first-line AZT/IFN achieved an overall response rate of 86% including 29% CR for unfavorable chronic ATL, 56% including 23% CR for acute ATL, and 33% including 16.5% CR for ATL lymphoma. Importantly, patients with aggressive ATL who achieved CR after AZT/IFN achieved a median PFS of 48 months as compared to 11 months for patients treated with chemotherapy (*P* = 0.003).

The mechanism of action of the combination of AZT and IFN remains poorly understood with arguments playing in favor of either a direct cytotoxic effect or an antiviral effect (Nasr et al., 2011). Kinpara et al. reported that IFN inhibited viral gene expression and induced cell cycle arrest in IL-2-dependent HTLV-1-infected T-cells. Furthermore, when combined with AZT, IFN triggered p53 signaling and cell apoptosis in these cells (Kinpara et al., 2013). On the other hand, AZT has been described to persistently inhibit telomerase resulting in reprogramming of HTLV-I-infected cells to p53-mediated senescence. These results likely explain the lower efficacy of AZT/IFN in ATL patients bearing p53 mutation (Datta et al., 2006). A recent report demonstrated that this combination inhibited the HTLV-1 reverse transcriptase activity and modified the viral clonality pattern in responding but not in resistant ATL patients (Macchi et al., 2017). Since reverse transcriptase-mediated viral replication does not occur in the malignant cells, these results highly suggest that the primary target of the AZT/IFN combination is the ATL microenvironment, specifically de novo infection of T cells by HTLV-1 which appears critical for the survival of the malignant clone.

Overall, these results have transformed the clinical management of ATL in most parts of the world. Indeed, AZT/IFN showed highly effective and significantly improved survival in the leukemic chronic and smoldering subtypes of ATL as well as in a subset of the acute subtype with wild-type P53. Patients with the lymphoma subtype benefited from induction chemotherapy, when given simultaneously or sequentially with AZT and IFN. Patients with previously untreated or newly diagnosed ATL achieved higher response rates compared to heavily treated patients. Unfortunately, many patients either are resistant or progress even after a long period of disease control. Furthermore, treatment should be continued for life as relapse always occurs upon stopping therapy, indicating that AZT/IFN is not curative. Finally, an ongoing prospective study in Japan is randomizing AZT/IFN versus watch and wait in patients with symptomatic smoldering or favorable chronic ATL (JCOG1111C).

### Combination Chemotherapy

#### Prognosis

The Lymphoma Study Group (LSG) of the Japan Clinical Oncology Group (JCOG) has analyzed prognostic factors for each subtype of ATL (Members of the Lymphoma Study Group, 1991; Takatsuki, 1994). In all patients with ATL, advanced age (40 years or greater), advanced performance status (PS), high LDH/calcium levels, and four or more involved lesions were unfavorable factors. These factors could be used to construct a model for risk grouping (Members of the Lymphoma Study Group, 1991). For patients with chronic-type ATL, the major prognostic factors were serum LDH, albumin, and blood urea nitrogen. Patients with chronic-type ATL and normal values for the three factors (30% of patients with chronic type disease) showed a prognosis as good as that of patients with smoldering-type ATL (Takatsuki, 1994). Thus, in Japan, patients with the favorable chronic type with normal LDH, albumin, and blood urea nitrogen values may not need to be treated immediately but are placed on follow-up without treatment, whereas patients with the unfavorable chronic type who have an abnormal value for at least one of the three factors that have an MST of 15 months are treated with cytotoxic chemotherapy (Takatsuki, 1994; Tsukasaki et al., 2009; Cook Lucy et al., 2019).

The International T-Cell Lymphoma Project found that ATL was the 4th most frequent PTCL, mainly in Japan, and the prognosis after therapy was worst among the lymphomas (Vose et al., 2008). The worst prognosis is associated with the multiagent chemoresistance of ATL cells, opportunistic infection, high tumor burden with multiple-organ involvement, complicated hypercalcemia, and advanced age at onset (Takatsuki, 1994; Tsukasaki et al., 2009).

Recently, a retrospective analysis of 807 patients in Japan revealed a prognostic index for acute/lymphoma-type ATL who did not receive allo-HSCT consisting of clinical stage, age, performance status, serum albumin, and serum IL-2R (Katsuya et al., 2012). This index was reproducible in the validation set, with median OS of 16.2, 7.3, and 3.6 months, respectively, for patients at low, intermediate, and high risks. JCOG-LSG conducted a meta-analysis of three consecutive trials only including aggressive ATL (see below) (Fukushima et al., 2014). Analysis of OS in a total of 276 patients with aggressive ATL revealed 2 significant prognostic factors: hypercalcemia and performance status. In the validation set, a proposed prognostic index using the 2 factors into 2 strata revealed median OS of 17.8 and 6.3 months, respectively, for patients at low or high risk. However, the 5-year OS rate was below 15% even in the low-risk group of both indexes, and hence the subgroup of patients with optimal prognosis could not be identified.

#### Clinical Trials by the Japan Clinical Oncology Group

Six consecutive chemotherapy trials focusing on ATL have been conducted by JCOG-LSG since 1978 (Shimoyama et al., 1982; Shimoyama et al., 1988; Yamada et al., 2001; Tsukasaki et al., 2003; Tsukasaki et al., 2007; Tsukasaki et al., 2012). The initial LSG1 study (1978–1980) applied VEPA, which consisted of a combination of vincristine (VCR), cyclophosphamide (CPA), prednisolone (PSL), and doxorubicin (DOX). Patients enrolled in this trial included advanced NHL and advanced-stage ATL. Whereas B-cell lymphoma achieved the highest CR followed by peripheral non-ATL T-cell lymphoma (PNTL), ATL patients had the lowest CR rate (64, 36, 18%, respectively) (Shimoyama et al., 1982). LSG1-VEPA was later compared to LSG2-VEPA with methotrexate (LSG2-VEPA-M) in NHL including advanced ATL patients recruited to a phase III clinical trial (1981–1983) (Shimoyama et al., 1988). PNTL patients were differentiated from those with ATL based on anti-HTLV-1 antibodies in their sera. Patients who received LSG2-VEPA-M had a better CR rate than those who were treated with LSG1-VEPA (37 and 17%, respectively; *P* = 0.09). In this study, B cell lymphoma and PNTL patients benefited better than ATL patients whose CR rate was significantly lower (*P* < 0.001). The MST of the 54 treated ATL patients given LSG1/2 was 6 months, and the 4-year OS rate was only 8%.

A phase II study (1987–1991) of second-generation combination chemotherapy against advanced aggressive NHL including ATL was conducted (JCOG8701). This multiagent chemotherapy, named LSG4, consisted of three different regimens VEPA-B-VCR, CPA, PSL, DOX, and bleomycin (BLM); M-FEPA-methotrexate (MTX), vindesine (VDS), CPA, PSL, and DOX; and VEPP-B-VCR, etoposide (ETP), procarbazine (PCZ), PSL, and BLM, and comprised nine agents in total (Tsukasaki et al., 2012). The CR for advanced NHL including ATL was higher than that of patients included in the LSG1/2 study (72 and 57%, respectively; *P* < 0.05). Although ATL patients had a worse CR rate as compared to B cell lymphoma and PNTL patients in this LSG4 study (*P* < 0.01), they showed a better CR rate in this trial compared to those enrolled in the previous LSG1/LSG2 studies (43 and 28%, respectively) but maintained a poor prognosis, with an MST of 8 months and a 12% 5-year OS rate. However, the continuous CR rate improved from 4% (2 of 54) in the LSG1/2 studies to 12% (5 of 43). A multivariate analysis of the aggressive NHL in JCOG8701 (*n* = 267) revealed the diagnosis of ATL as the worst prognostic factor (relative risk: 3.2; *P* = 0.0001) in Japan (Tsukasaki et al., 2012).

The poor results in ATL with conventional chemotherapies necessitated the investigation of novel agents. Phase I and II trials of 2′-deoxycoformycin (DCF; pentostatin, an irreversible inhibitor of adenosine deaminase) were performed for ATL in Japan. The phase II study showed an overall response rate of 32% (10 of 31) in relapsed/refractory ATL (2 CRs and 8 PRs) (Tobinai et al., 1992). These promising findings and the proposal of subtype classification of ATL (see above and Table 1) urged the Japanese researchers to initiate a DCF-containing phase II study (JCOG9109; LSG11) exclusively against patients with untreated aggressive ATL since 1991 (Tsukasaki et al., 2003). Sixty-two patients (34 patients with acute, 21 with lymphoma, and 7 with unfavorable chronic subtypes) were enrolled. VCR, DOX, ETP, PSL, and DCF were administered every 28 days for 10 cycles until progressive disease or severe toxic complication. Among the 61 patients who were evaluable for toxicities, four patients (7%) developed treatment-related death (TRD). In the 60 eligible patients, 17 (28%; 95% CI: 19% to 41%) achieved CR, while 14 achieved PR (response rate: 52%; 95% CI: 39% to 64%). After a median observation time of 27 months, the MST was 7.4 months, and the OS rate at 2 years was 17%, similar findings to the results with the previous LSG4 (JCOG8701).

A subsequent phase II study (JCOG9303; LSG15) was initiated in 1994 to study a nine-agent combination regimen consisting of VCR, CPA, DOX, PSL, ranimustine (MCNU), VDS, ETP, and carboplatin (CBDCA) with MTX and PSL intrathecally administrated, for untreated patients with aggressive ATL (Yamada et al., 2001). In this study, the relative dose intensification was associated with the prophylactic use of the granulocyte colony-stimulating factor (G-CSF). Furthermore, non-cross-resistant drugs such as MCNU and CBDCA were incorporated into the regimens. Acute (*n* = 58), lymphoma (*n* = 28), and unfavorable chronic-type ATL patients (*n* = 10) were enrolled in this study. Among the ninety-three eligible patients, response was achieved in 81% (75 pts), with 33 patients achieving CR and 42 PR (35 and 45%, respectively). The 2-year OS rate of all eligible patients (*n* = 93) was 31%. MST was 13 months, and the median follow-up duration of the 20 surviving patients was 4.2 years. Regarding the adverse effects, only one patient experienced grade 4 non-hematological toxicity whereas 65% and 53% of the patients experienced Grade 4 hematological toxicities, mostly neutropenia and thrombocytopenia.

To confirm the clinical efficacy of LSG15 as novel standard treatment for aggressive ATL, a first randomized phase III (JCOG9801) study between mLSG15 and CHOP-14 (CPA, DOX, VCR, and PSL) was initiated in 1998 (Tsukasaki et al., 2007). Newly diagnosed patients with aggressive ATL were randomly assigned to receive one of the following regimens: either six courses of LSG15 every four weeks or eight courses of CHOP-14 both combined with G-CSF and intrathecal administration (ITA). mLSG15 in JCOG9801 was a modified version of LSG15 in JCOG9303, consisting of three regimens, VCAP [VCR, CPA, ADM, PSL] on day 1; AMP [ADM, MCNU, PSL] on day 8; and VECP [VDS on day 15, ETP on days 15 to 17, CBDCA on day 15, PSL on days 15 to 17] on days 15–17, and the next course to be initiated on day 29. The modified mLSG15 protocol allowed a reduction in the total number of cycles (6 instead of 7) to overcome progressive thrombocytopenia following the repeated administration of the VCAP-AMP-VECP therapy as well as the addition of cytarabine to MTX and PSL for prophylactic ITA, at the recovery phases to overcome the frequent central nervous system (CNS) relapse observed in the JCOG9303 trial. Among the 118 patients randomized in this mLSG15 trial, response was achieved in 72% with 23 patients achieving CR and 18 PR (40 and 32%, respectively). The ORR was 66%, with 15 patients achieving CR (25%) and 25 PR (41%) in the CHOP-14 regimen. CR rate significantly improved with the mLSG15 group as compared to the CHOP-14 group (40% vs. 25%, respectively; *p* = 0.02). This was accompanied with a better median PFS time and 1-year PFS (7.0 months and 28% with mLSG15 and 5.4 months and 16% in the CHOP-14 regimen, respectively; *p* = 0.10). The MST and 3-year OS slightly improved (12.7 months and 24% in mLSG15 and 10.9 months and 13% in CHOP-14; *p* = 0.085). After adjusting the clinical characteristics by Cox regression, the *P*-value for OS became 0.029 because of uneven prognostic factors such as bulky masses and B symptoms. More adverse effects were noted, in the mLSG15 vs. CHOP-14. 3 TRDs were reported in addition to a higher percentage of patients who presented with grade 4 neutropenia, grade 4 thrombocytopenia, and grade 3–4 infection in mLSG15 as compared to CHOP-14 (98% vs. 83%, 74% vs. 17%, and 32% vs. 15%, respectively). These findings led to the conclusion that mLSG15 is more effective than CHOP-14 and led to improved 3-year OS and CR rate, but this was at the expense of higher toxicity, prompting further research for the treatment of aggressive ATL whose prognosis is still poor as compared to other hematological malignancies. Allo-HSCT is now applied for the treatment of young patients with aggressive ATL (see below). To confirm the efficacy of allo-HSCT, especially in view of a comparison with a historical control of intensive chemotherapy (mLSG15 chemotherapy in JCOG9801), a confirmatory phase II study of mLSG15 chemotherapy and upfront allo-HSCT is ongoing (JCOG0907).

#### Treatment and Prevention of Complications (Tsukasaki et al., 2009)

Hypercalcemia, which is a frequent complication of aggressive ATL, usually can be controlled with cytotoxic therapy and the appropriate use of other calcium-lowering agents, supplementary liquids, and diuretics even when the PS of the patient is poor.

Opportunistic infections frequent in HIV-infected individuals are also frequent in ATL patients. Early detection and intervention of the infections are mandatory before or concomitantly to the chemotherapy of ATL.

Patients with ATL require some supportive or preventive treatment for fungal, protozoal, and viral infections. A low dose of cotrimoxazole and an oral antifungal agent are recommended, together with cytotoxic therapy. An anti-strongyloides drug, such as ivermectin or albendazole, should be considered to avoid systemic infections in patients with a history of exposure to the parasite in tropical regions.

There are case reports of B-cell NHL associated with Epstein–Barr virus and of Kaposi’s sarcoma in patients with ATL (Tsukasaki et al., 2009). The profound immunodeficient state in patients with ATL might allow the emergence of such opportunistic tumors.

### Allo-HSCT

Allo-HSCT is an important curative treatment option in aggressive ATL patients, contrary to autologous HSCT (Tsukasaki et al., 1999). Although some patients with aggressive ATL who achieve complete remission after AZT/IFN therapy or after intensive chemotherapy may achieve a long PFS (>5 years), most patients with aggressive ATL succumb to the disease (Bazarbachi et al., 2010; Fukushima et al., 2014). Since multiple retrospective studies of allo-HSCT in ATL patients reported a favorable outcome (Utsunomiya et al., 2001; Fukushima et al., 2005; Kato et al., 2007; Hishizawa et al., 2010), the number of patients receiving allo-HSCT has been constantly increasing. By 2018, more than 2000 allo-HSCTs were performed in Japan for ATL patients. Although most allo-HSCT outcomes have been reported in Japanese ATL patients, the European Society for Blood and Marrow Transplantation’s Lymphoma Working Party has also shown similar results (Bazarbachi et al., 2014). Thus, allo-HSCT can provide a chance of long-term remission through a graft-versus-ATL (GvATL) effect, and upfront allo-HSCT has been recommended for transplant-eligible patients with aggressive-type ATL (Fuji et al., 2018; Cook Lucy et al., 2019).

#### Donor Sources

**FIGURE 1:**
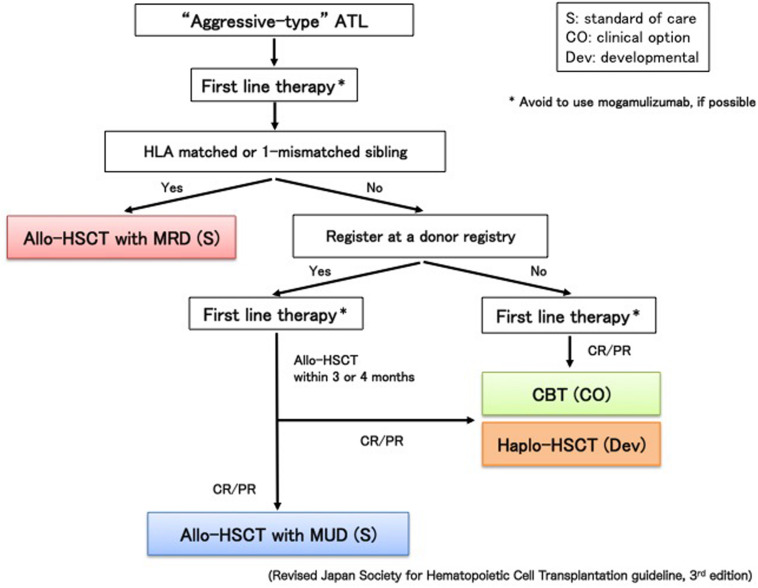
Algorithm for donor selection in allo-HSCT. Match-related donor (MRD), hematopoietic stem cell transplantation (HSCT) and cord blood transplantation (CBT).

Because prognosis of ATL patients with progressive disease at transplantation is dismal and responses to chemotherapy in patients with aggressive-type ATL are not durable, searching for a human leukocyte antigen (HLA)-matched related donor (MRD) or an HLA-matched unrelated donor (MUD) at diagnosis is recommended for the appropriate timing of upfront allo-HSCT (Figure 1); however, availability of MRD and MUD is limited. In addition, allo-HSCT from alternative donor sources earlier in better disease status may increase the potential to improve outcomes (Fuji et al., 2016a). Therefore, the number of ATL patients receiving cord blood transplantation (CBT) or haploidentical HSCT (haplo-HSCT) has recently increased.

According to donor sources, OS at two years in ATL patients who received allo-HSCT with MRD or MUD was approximately 40%, whereas OS at 2 years with UCB showed 20% (Hishizawa et al., 2010; Kato et al., 2014). It is certainly difficult to directly compare outcomes between different donor sources because the graft source selection is strongly influenced by donor availability. However, retrospective studies of CBT and haplo-HSCT in ATL patients reported unsatisfactory results (Hishizawa et al., 2010; Kato et al., 2014; Yoshimitsu et al., 2018). Prospective clinical trials using CBT or haplo-HSCT with post-cyclophosphamide have been performed (Cook Lucy et al., 2019), although the efficacy of alternative donor sources in ATL is not known.

The selection of HTLV-1 carriers as donors for ATL patients has been controversial. The updated report showed that there was no significant difference in OS or cumulative incidence of either relapse or transplantation-related mortality (TRM) between HTLV-1-seropositive- and HTLV-1-seronegative-related donors (Yoshimitsu et al., 2019). Selecting HTLV-1-seropositive donors might not be contraindicated for ATL patients; however, the recent International Consensus Meeting Report preferred the use of HTLV-1-seronegative donors in allo-HSCT for ATL patients to avoid the risk of donor cell-derived ATL after transplantation (Tamaki and Matsuoka, 2006; Cook Lucy et al., 2019). If only HTLV-1-seropositive-related donors are available, it is recommended that no monoclonal or oligoclonal integration by Southern blot analysis of HTLV-1 proviral DNA is required for the selection as a donor.

#### Conditioning

Similar to other hematological malignancies, both myeloablative conditioning (MAC) and reduced-intensity conditioning (RIC) have been used in patients with ATL. However, early experience of allo-HSCT following MAC in ATL patients was unsatisfactory, with approximately 40–50% of higher TRM rate. In addition, the recent median age of ATL at diagnosis was reported to be 68 years in Japan (Nosaka et al., 2017). Hence, RIC regimens have been increasingly used. In the retrospective analysis of the Japanese nationwide survey, the OS rates were not different in ATL patients receiving allo-HSCT with either MAC or RIC (Ishida et al., 2012a). The prospective studies showed that RIC was also feasible and effective for elderly patients with aggressive-type ATL. Okamura et al. first reported that the 5-year OS of allo-HSCT with MRD was 34% (Okamura et al., 2005). Other prospective trials assessing RIC followed by allo-HSCT with MUD or CBT were performed (Cook Lucy et al., 2019). Intensity of conditioning should be considered by patient’s age, disease status, and performance status at transplantation.

#### Transplantation-Related Mortality and Relapse

Although the prognosis of patients with ATL has been apparently improved with treatment by allo-HSCT in retrospective studies, survival rates themselves of allo-HSCT have not dramatically improved during the last decade. The high TRM rate of ATL patients (approximately 30%–40%) has been problematic. ATL patients are generally immunocompromised and have frequent complications of various infections following allo-HSCT (Itonaga et al., 2013a). A high incidence of CMV infection was reported (Nakano et al., 2014), and CMV infection has been associated with poor prognosis in patients with ATL who received allo-HCT (Sawayama et al., 2019). The impact of infectious complications after allo-HSCT in ATL patients is different from that in patients with other hematological malignancies. More intensive care for infection is required to reduce TRM in ATL patients who receive allo-HSCT.

The high relapse rate of 30%–40% after transplantation has been also problematic. Disease status at transplantation remains a major risk factor for survival (Inoue et al., 2018). As discussed later, new targeted therapies including anti-CC chemokine receptor 4 (CCR4) antibody, mogamulizumab, and lenalidomide have been recently approved in Japan for ATL patients (Yamamoto et al., 2010; Ishida et al., 2012b; Ishida et al., 2016; Ogura et al., 2016). However, aggressive ATL patients who received mogamulizumab prior to allo-HSCT had a significantly increased risk of severe and steroid-refractory graft-versus-host disease (GVHD) likely because of depleting CCR4-positive regulatory T cells as well as ATL cells (Fuji et al., 2016b; Sugio et al., 2016). Therefore, when upfront allo-HSCT is planned, mogamulizumab should not be used during induction. A minimum of 50 days between the last dose of mogamulizumab and allo-HSCT is recommended, and additional intensification of GVHD prophylaxis should be considered (Cook Lucy et al., 2019).

The prognosis of ATL patients who relapse after allo-HSCT is extremely poor with a two-year OS of around 10%. Nevertheless, some long-term survivors have been reported following tapering and withdrawal of immunosuppression (Kato et al., 2019). Patients who could receive DLI had better survival, suggesting the existence of GvATL even after relapse (Itonaga et al., 2013b; Kato et al., 2019). Therefore, further studies on the improvement of the prognosis of patients with ATL who relapse after allo-HSCT would be worthwhile focusing on how GvATL is enhanced. Mogamulizumab may induce not only a strong cytotoxic effect for CCR4-positive ATL cells but also enhancement of a GvATL effect through the decrease in normal regulatory T-cells. Lenalidomide also has an immunomodulatory effect, possibly to enhance a GvATL effect. Preemptive treatment should be considered following the detection of minimal residual disease after transplantation, although methods of monitoring for minimal residual disease of ATL have not been well established. Previous reports showed that the changes in HTLV-1 proviral load (PVL) were various after allo-HSCT, and some patients had positive PVL despite CR and full donor chimerism (Choi et al., 2011). Reports regarding the treatment strategy for relapse after allo-HSCT remain limited.

### Approved New Agents

Two novel drugs, mogamulizumab, an anti-CCR4 monoclonal antibody (mAb), and lenalidomide, an immunomodulatory agent, have been approved for ATL recently in Japan following pivotal studies for relapsed aggressive ATL (Yamamoto et al., 2010; Ishida et al., 2012b; Ishida et al., 2016; Ogura et al., 2016). The former has also been approved for aggressive ATL as initial treatment combined with multiagent chemotherapy (Ishida et al., 2015). In addition, brentuximab vedotin (Bv) was approved for the initial treatment of CD30+ PTCL including CD30 + ATL.

Previous reports have demonstrated a high expression of CCR4 on ATL cells in ∼90% of patients as well as CCR4 mutation in 26% of ATL cases (Ishida et al., 2003; Nakagawa et al., 2014). Both aberrantly expressed Fra-2 and HBZ induce CCR4 expression and promote ATL cell proliferation (Nakayama et al., 2008; Sugata et al., 2016). Phase I and II clinical studies performed in Japan tested the efficacy of mogamulizumab, an anti-CCR4 defucosylated humanized mAb with higher ADCC activity, and reported an overall response rate of around 50%. The toxicity profile was acceptable, ranging from moderate to severe cutaneous and other immunopathology reactions (Yamamoto et al., 2010; Ishida et al., 2012b). Response rates were different between target lesions with high CR rates in peripheral blood compared to intermediate and low CR rates in cutaneous lesion and lymph node, respectively. Mogamulizumab treatment produced a median PFS of 5.2 months and OS of 13.7 months (Ishida et al., 2012b). A randomized phase II study in untreated aggressive ATL demonstrated a higher CR rate [52% (95% CI, 33–71)] for the combination of mLSG15 with mogamulizumab as compared to mLSG15 alone [33% (95% CI, 16–55)] (Ishida et al., 2015). The low sample size and short follow-up period were possibly the reason why PFS and OS were identical in both treatment groups. In the transplant setting, reports from large retrospective studies showed higher fatal GvHD after allogeneic stem cell transplant in aggressive ATL patients treated with mogamulizumab and chemotherapy before transplant as compared to those who received chemotherapy alone. Accordingly, mogamulizumab is not recommended pre allo-HSCT (Fuji et al., 2016b; Inoue et al., 2016; Sugio et al., 2016). Finally, a multi-country randomized phase II study was initiated to study the efficacy of mogamulizumab as a single treatment as compared to the investigators’ selection of salvage chemotherapy for relapsed/refractory aggressive ATL and revealed a better ORR in the mogamulizumab arm vs the investigator’s selection (15% vs 0%, respectively) (Phillips et al., 2019).

A phase I trial in patients with relapsed ATL or PTCL demonstrated the safety and preliminary antitumor activity of lenalidomide but at a higher dose than multiple myeloma (Ogura et al., 2016). Later on, in a phase II study of lenalidomide monotherapy in patients with relapsed aggressive ATL, responses were achieved in 11 of 26 patients, including 4 CR and 1 unconfirmed CR (Ishida et al., 2016). The median PFS and OS were 3.8 and 20.3 months, respectively. Lenalidomide achieved responses in CNS involvement by diffuse large B-cell lymphoma, providing a rationale for its use in aggressive ATL despite the lack of data because CNS involvement was an exclusion criterion in the phase 1 and 2 studies for ATL (Cook Lucy et al., 2019).

CD30 expression is variable in ATL, with a few % to modest in most ATL cases and, similar to anaplastic large cell lymphoma, with 100% in a few % of ATL cases (Ohshima et al., 2017). Brentuximab vedotin (Bv) is an antibody–drug conjugate composed of an anti-CD30 monoclonal antibody conjugated by a protease-cleavable linker to the microtubule-disrupting drug monomethyl auristatin E. Based on the promising activity and manageable toxicity profile observed in a phase 1 trial (Fanale et al., 2014) combining Bv with cyclophosphamide, doxorubicin, and prednisone (CHP [CHOP without vincristine] to eliminate the risk of overlapping neurotoxicity that could be worsened by delivering two microtubule-disrupting drugs), the double-blinded phase 3 global trial was initiated to compare BvCHP with standard CHOP for the treatment of previously untreated patients with CD30-positive (≥10% of cells by local review) PTCL (Horwitz et al., 2019). Four hundred and fifty-two patients with PTCL were enrolled, and 226 patients were randomly assigned to both the BvCHP and the CHOP arms. Median PFS was 48⋅2 months (95% CI 35⋅2–not estimable) in the BvCHP arm and 20⋅8 months (12⋅7–47⋅6) in the CHOP arm (HR 0⋅71 [95% CI 0⋅54–0⋅93], *p* = 0⋅0110). Adverse events, including incidence and severity of febrile neutropenia (41 [18%] patients in the BvCHP arm and 33 [15%] in the CHOP arm) and peripheral neuropathy (117 [52%] in the BvCHP arm and 124 [55%] in the CHOP arm), were similar between arms. Based on the results, BvCHP was approved for the initial treatment of CD30+ PTCL. However, most of the enrolled patients were with anaplastic large cell lymphoma (316 patients), and those with PTCL, not otherwise specified, angioimmunoblastic T cell lymphoma, ATL, and enteropathy-associated T cell lymphoma were 72, 54, 7, and 3, respectively.

### Prevention

Mother-to-infant transmission through breastfeeding, sexual transmission, and parenteral transmission through blood transfusion or intravenous drug use are the main risk factors for HTLV-I infection. Accordingly, there are two major steps for the prevention of HTLV-1-associated ATL (Tsukasaki et al., 2009). The first is the prevention of HTLV-1 infections. This has been modestly established in Japan by screening for HTLV-1 among blood donors and pregnant women, discarding the HTLV-1 positive blood and recommending the mothers who are found to be carriers to refrain from breastfeeding, respectively (Tsukasaki et al., 2009; Satake et al., 2016). For several decades, before starting the interventions, the prevalence of HTLV-1 has declined drastically in Japanese endemic areas, probably because of birth cohort effects. The second is the prevention of ATL development among HTLV-1 carriers. This has not been achieved partly because only about 5% of HTLV-1 carriers develop the disease in their lifetime although several risk factors have been identified, so-called high-risk HTLV-1 carriers (Iwanaga et al., 2010). Furthermore, no agent has been found to be effective in preventing the development even in the high-risk carriers (Tsukasaki et al., 2009).

## Conclusion

Various treatment options are applicable for ATL based on subtype classification, consisting in Japan a watchful waiting for indolent ATL and intensive chemotherapy followed by allo-HSCT for aggressive ATL and in many parts of the world a combination of IFN and AZT for indolent ATL or patients with leukemic manifestation. Several new drugs, including mogamulizmab, lenalidomide, and brentuximab vedotin, have been approved for ATL after clinical trials. Furthermore, several promising new agents and treatment modalities are now undergoing clinical studies associated with translational research (see section “Therapeutic Strategies of ATL Based on Its Clinical Subtypes”).

## Author Contributions

All authors listed have made a substantial, direct and intellectual contribution to the work, and approved it for publication.

## Conflict of Interest

The authors declare that the research was conducted in the absence of any commercial or financial relationships that could be construed as a potential conflict of interest.
